# Thick-wall cavity predicts worse progression-free survival in lung adenocarcinoma treated with first-line EGFR-TKIs

**DOI:** 10.1186/s12885-018-4938-9

**Published:** 2018-10-23

**Authors:** Fei Zhou, Wanrong Ma, Wei Li, Huijuan Ni, Guanghui Gao, Xiaoxia Chen, Jie Zhang, Jingyun Shi

**Affiliations:** 1 Department of Oncology, Shanghai Pulmonary Hospital, Tongji University School of Medicine, No 507 Zhengmin Road, Yangpu District, Shanghai, China; 2 Department of Intensive Care Unit, Shanghai Jingan District Shibei Hospital, Shanghai, China; 3 Department of Imaging, Shanghai Pulmonary Hospital, Tongji University School of Medicine, No 507 Zhengmin Road, Yangpu District, Shanghai, 200433 China

**Keywords:** Cavitation, Adenocarcinoma, EGFR-TKI, Hypoxia

## Abstract

**Background:**

Cavity occurs in 5.7 to 14.9% of patients with lung adenocarcinoma (ADC). However, the impact of cavity on the therapeutic response to epidermal growth factor receptor-tyrosine kinase inhibitors (EGFR-TKIs) in ADC patients with *EGFR* mutations remains unclear. The aim of the present retrospective study was to elucidate the incidence and detailed characteristics of *EGFR*-mutant cavitary ADC and investigate the efficacy of EGFR-TKI treatment in this subgroup.

**Methods:**

Two hundred seventy-six consecutive patients with advanced *EGFR*-mutant lung ADC treated with first-line EGFR-TKIs were enrolled. Cavitation and the thickness of cavity wall were assessed based on high-resolution computed tomography scans. Progression-free survival (PFS) was analyzed by the Kaplan-Meier plots and the log-rank test was used to calculate the significance between groups.

**Results:**

Cavity occurred in 5.4% (15/276) of patients with *EGFR*-mutant lung ADC and was more prevalent among male patients (66.7% vs. 33.3%, *P* = 0.008). Of the 15 *EGFR*-mutant cavitary ADC, 9 patients had exon 19 deletion (19DEL) and 6 harbored L858R mutation, 9 patients had thick-wall cavity while 6 had thin-wall cavity. Cavity had an adverse impact on the PFS of *EGFR*-mutant ADC treated with first-line EGFR-TKIs (noncavity versus cavity, 11.0 versus 6.5 months, hazard ratio [HR]: 0.33, 95% confidence interval [CI], 0.15–0.73, *P* = 0.003). The impaired effect was only observed in patients with L858R mutation (11.0 vs. 4.2 months, HR: 0.05, 95%CI, 0.01–0.27, *P* = 0.0003) but not in those with 19DEL (10.4 versus 9.7 months, HR: 0.73, 95%CI, 0.30–1.75, *P* = 0.483). All six L858R-mutant cavitary ADC patients had thick-wall cavity while thick-wall cavity was only identified in one thirds (3/9) of patients with 19DEL. Further analyses showed that patients with thick-wall cavity had worse PFS (6.0 versus 11.0 months, *P* = 0.013). Multivariate analysis identified cavity as an independent predictive factor for PFS (HR: 0.49, 95% CI, 0.26–0.90, *P* = 0.022).

**Conclusion:**

Cavitary ADC was associated with a worse PFS of first-line EGFR-TKI therapy, mainly in those with L858R mutation. Thick-wall cavity formation may be the main cause that contribute to the worse PFS.

## Background

Epidermal growth factor receptor (*EGFR*) sensitizing mutations occur in about 40–78% of Asian patients with lung adenocarcinoma (ADC) [1–4]. For patients with *EGFR* sensitizing mutations, EGFR-tyrosine kinase inhibitors (EGFR-TKIs) have resulted in a significant improvement in progression-free survival (PFS), objective response rate (ORR), and quality of life (QoL) when compared with classical platinum-based chemotherapy [5–8]. However, even in *EGFR*-mutant patients, 10–30% may not benefit from initial EGFR-TKI treatment and even experience rapid disease progression [5–8].

Cavitation in a tumor nodule is previously thought to be more prevalent in patients with squamous cell carcinoma [9, 10]. As the prevalence of lung ADC increases, cavitary ADC has also been reported, with an incidence of 5.7 to 14.9% in patients with lung ADC [11–14]. Compared with noncavitary ADC, cavitary ADC is associated with a worse prognosis [14]. Previous study has demonstrated that inadequate vascularization might account for cavity formation and cavitary ADC has a higher frequency of vascular invasion than noncavitary ADC [14]. Therefore, the occlusion of feeding vessels by vascular invasion in cavitary ADC could create a hypoxia microenvironment. As hypoxia could induce resistance to EGFR-TKIs in *EGFR*-mutant lung cancer [15, 16], we speculate cavity formation may impair the therapeutic response to EGFR-TKIs in *EGFR*-mutant ADC patients.

The aim of the present retrospective study was to elucidate the incidence and detailed characteristics of *EGFR*-mutant cavitary ADC and investigate the efficacy of EGFR-TKI treatment in this subgroup.

## Methods

### Patient selection

Consecutive patients with advanced *EGFR*-mutant lung ADC treated with first-line EGFR-TKIs at the Department of Oncology, Shanghai Pulmonary Hospital, China from November 2011 to January 2016 were enrolled. The lung ADC diagnosis was diagnosed pathologically according to World Health Organization (WHO) pathology classification [17]. All clinicopathologic data were extracted from electronic medical record in Shanghai Pulmonary Hospital. *EGFR* common mutations were defined as mutations including exon 19 deletion (19DEL) and Leu858Arg point mutation in exon 21 (L858R). *EGFR* rare mutations were those mutations in exons 18 and 20, other than 19DEL and L858R mutations.

The study protocol was approved by the Ethics Committee of Shanghai Pulmonary Hospital. The written informed consent was obtained from each participant to use the clinical data for research before any medical interventions.

### Review of computed tomography images

Computed tomography (CT) scans were performed for all patients via two CT machines (Brilliance, Philips Medical Systems Inc., Cleveland, the US [64 × 1 mm acquisition; slice width 1 mm] or SOMATOM Definition AS, Siemens Aktiengesell-schaft, Munich, Germany [128 × 1 mm acquisition; slice width 1 mm]) before bronchoscopy or a percutaneous CT-guided biopsy.

The CT images were evaluated by two investigators (FZ and WL) for tumor cavitation, independently. Tumor cavitation was defined as the presence of an air-containing space with a diameter of greater than 5 mm within the primary tumor and which was not identifiable as an airway, as previous described [14, 18]. Disagreements were resolved by consensus or a third reviewer (JZ or JS).

The thickness of cavity wall was measurable at intervals of 1 mm, which was determined based on the thickest segment of the cavity wall totally orthogonal to the image plane. According to previous study [18], a cavity wall thickness of greater than 4 mm was defined as thick-wall cavity while a cavity wall thickness 4 mm or less was defined as thin-wall cavity. The dynamic volume perfusion CT (dVPCT) was used to quantitatively assess tumor permeability, blood flow (BF), blood volume (BV) and mean transit time (MTT). The detailed procedures of dVPCT were described in our previous studies [19, 20].

### Molecular analyses

All mutational analyses were performed at the Department of Lung Cancer and Immunology, Shanghai Pulmonary Hospital. Briefly, DNA from tumor tissue was extracted using the DNeasy Blood and Tissue Kit or the QIAamp DNA FFPE Tissue Kit (both from Qiagen, Hilden, Germany). *EGFR* mutations (exons 18–21) were detected by amplification refractory mutation system (ARMS) (Amoy Diagnostics Co. Ltd., Xiamen, China). At the time of development of acquired resistance, re-biopsy samples were obtained from either primary sites or metastasis sites for further analysis to identify potential mechanisms. Detailed procedures were described in our previous studies [21–24].

### Statistical analysis

Categorical variables were compared using Fisher’s exact test or Chi-square test, and continuous variables were compared using the Mann–Whitney U test. PFS was defined as the time from treatment commencement of EGFR-TKI to confirmed disease progression or death of any cause. PFS was analyzed by the Kaplan-Meier plots and the log-rank test was used to calculate the significance between groups. The predictive factors for PFS were analyzed using univariate and multivariate COX proportional hazard model. The two-sided significance level was set at *P* < 0.05. Data were analyzed using the Statistical Package for the Social Sciences Version 23.0 Software (SPSS, Inc., Chicago, IL) and the survival curve was drawn with GraphPad Prism 5.01 (GraphPad Software, San Diego, CA).

## Results

### Patient characteristics

Two hundred and seventy-six patients were evaluated for the present study. Cavitary ADC was identified in 5.4% (15/276) of patients with *EGFR*-mutant lung ADC. The median age of cavitary ADC was 61 years (range, 37 to 76). Specifically, cavitary ADC was more common in male patients when compared with noncavitary ADC (66.7% vs. 33.0%, *P* = 0.008). The tumor size of cavitary ADC was marginally smaller than that of noncavitary ADC (2.87 vs. 3.76 cm, *P* = 0.056). As shown in Table 1, there was no significant difference between cavitary and noncavitary ADC in terms of smoking history, ECOG PS, TNM stage, the incidence of brain, liver and bone metastases, types of *EGFR* mutations, and types of EGFR-TKIs received.

**Table 1:** Patient Characteristics in Cavitary and Noncavitary Adenocarcinoma with *EGFR* mutations

Characteristic	All patients *N* = 276	Cavity *N* = 15	Noncavity *N* = 261	*P*
Age, Median (range), y	61 (26–86)	61 (37–76)	61 (26–86)	0.271
≤65	178 (64.5)	12 (80.0)	166 (63.6)	
> 65	98 (35.5)	3 (20.0)	95 (36.4)	
Gender, no. (%)				0.008
Male	96 (34.8)	10 (66.7)	86 (33.0)	
Female	108 (65.2)	5 (33.3)	175 (67.0)	
Smoking history, no. (%)				0.494
Nonsmokers	224 (81.2)	11 (73.3)	213 (81.6)	
Former or current smokers	52 (18.8)	4 (26.7)	48 (18.4)	
ECOG PS, no. (%)				0.377^a^
0	10 (3.6)	1 (6.7)	9 (3.4)	
1	242 (87.7)	14 (93.3)	228 (87.4)	
2	20 (7.2)	0 (0.0)	20 (7.7)	
3	4 (1.4)	0 (0.0)	4 (1.5)	
TNM stage, no. (%)				0.478^b^
Recurrent	14 (5.1)	1 (6.7)	13 (5.0)	
IIIB	31 (11.2)	0 (0.0)	31 (11.9)	
IV	231 (83.7)	14 (93.3)	217 (83.1)	
T stage, no. (%)				0.270
T1–2	101 (36.6)	5(33.3)	96 (36.8)	
T3–4	175 (63.4)	10 (66.7)	165 (63.2)	
N stage, no. (%)				0.532
N0–1	64 (23.2)	2 (13.3)	62 (23.8)	
N2–3	212 (76.8)	13 (86.7)	199 (76.2)	
Mean tumor size (cm), ±SD	3.73 ± 1.74	2.87 ± 0.99	3.76 ± 1.76	0.056
Brain metastasis, no. (%)	71 (25.7)	4 (26.7)	67 (25.7)	1.000
Liver metastasis, no. (%)	17 (6.2)	1 (6.7)	16 (6.1)	1.000
Bone metastasis, no. (%)	116 (42.0)	8 (53.3)	108 (41.4)	0.362
EGFR-TKI, no. (%)				0.537^c^
Gefitinib	185 (67.0)	9 (60.0)	176 (67.4)	
Erlotinib	45 (16.3)	2 (13.3)	43 (16.5)	
Icotinib	44 (15.9)	4 (26.7)	40 (15.3)	
Afatinib/Osimertinib	2 (0.8)	0 (0.0)	2 (0.8)	
*EGFR* mutations, no. (%)				0.362^d^
Exon 19 deletion	117 (42.4)	9 (60.0)	108 (41.4)	
L858R mutation	130 (47.1)	6 (40.0)	124 (47.5)	
Rare^e^	29 (10.5)	0 (0.0)	29 (11.1)	

*Abbreviations: EGFR-TKI* epidermal growth factor receptor- tyrosine kinase inhibitor, *ECOG PS* Eastern Corporation Oncology Group performance status, *SD* standard deviation

^a^ECOG PS 0 or 1 vs. 2 or 3

^b^Recurrent/IIIB vs. Stage IV

^c^Gefitinib vs. Other EGFR-TKIs

^d^Exon 19 deletion vs. others

^e^including *EGFR* mutations in exons 18 and 20, other than 19DEL and L858R mutations

### Characteristics of the cavitary ADC patients with *EGFR* mutations

Of the 15 cavitary ADC patients with *EGFR* mutations, 10 were male and 5 were female, 11 were never-smokers and 4 were former or current smokers. Fourteen patients had stage IV disease and 1 had recurrent disease. Regarding mutational status, 9 patients had 19DEL and 6 harbored L858R mutation. All patients received first-generation EGFR-TKI as initial treatment, including 9 who received gefitinib, 2 who received erlotinib, and 4 who received icotinib. Regarding wall thickness of the cavity, 9 patients had thick-wall cavity while 6 had thin-wall cavity. When acquired resistance develops, 10 patients provided tumor tissue for evaluating the mechanisms of acquired resistance. The proportion of T790 M mutation was 40% (4/10) in overall group, 25% (1/4) in L858R mutation group, and 50% (3/6) in 19DEL group. The detailed characteristics of the cavitary ADC patients with *EGFR* mutations are listed in Table 2.

**Table 2:** Characteristics of the 15 Cavitary Adenocarcinoma patients with *EGFR* mutations

Patient No.	Age, y	Gender	Smoking	Stage	Tumor size	Wall	EGFR mutations	EGFR-TKI	PFS, mo	Efficacy	T790 M
1	62	Male	Never	IV	2.6 cm	Thick	L858R mutation	Erlotinib	4.7	SD	Unknown
2	71	Male	Never	IV	3.6 cm	Thick	L858R mutation	Gefitinib	3.3	PR	Negative
3	52	Female	Never	IV	2.2 cm	Thick	L858R mutation	Gefitinib	6.0	PR	Negative
4	44	Male	Never	IV	2.4 cm	Thick	L858R mutation	Gefitinib	9.6	PR	Positive
5	57	Male	Never	IV	2.0 cm	Thick	L858R mutation	Gefitinib	1.0	PD	Negative
6	50	Male	Smoker	IV	2.6 cm	Thick	L858R mutation	Erlotinib	4.2	SD	Unknown
7	76	Female	Never	IV	5.0 cm	Thick	Exon 19 deletion	Gefitinib	5.2	SD	Unknown
8	37	Female	Never	IV	2.8 cm	Thin	Exon 19 deletion	Icotinib	11.4	PR	Unknown
9	75	Male	Never	IV	2.2 cm	Thin	Exon 19 deletion	Icotinib	11.0	PR	Positive
10	61	Female	Never	IV	2.2 cm	Thin	Exon 19 deletion	Gefitinib	24.5	PR	Positive
11	47	Male	Smoker	IV	4.5 cm	Thick	Exon 19 deletion	Gefitinib	6.7	PR	Negative
12	48	Male	Never	IV	2.2 cm	Thick	Exon 19 deletion	Gefitinib	6.5	SD	Negative
13	51	Male	Smoker	IV	4.8 cm	Thin	Exon 19 deletion	Icotinib	9.7	SD	Negative
14	56	Female	Never	Recurrent	2.0 cm	Thin	Exon 19 deletion	Gefitinib	4.5	PR	Positive
15	39	Male	Smoker	IV	2.5 cm	Thin	Exon 19 deletion	Icotinib	3.0	SD	Unknown

*Abbreviations: EGFR-TKI* epidermal growth factor receptor- tyrosine kinase inhibitor, *ECOG PS* Eastern Corporation Oncology Group performance status, *SD* standard deviation, *PR* partial response, *SD* stable disease, *PD* progressive disease, *PFS* median progression-free survival

### Therapeutic responses to EGFR-TKI treatment in cavitary and noncavitary ADC patients with *EGFR* mutations

**Fig. 1:**
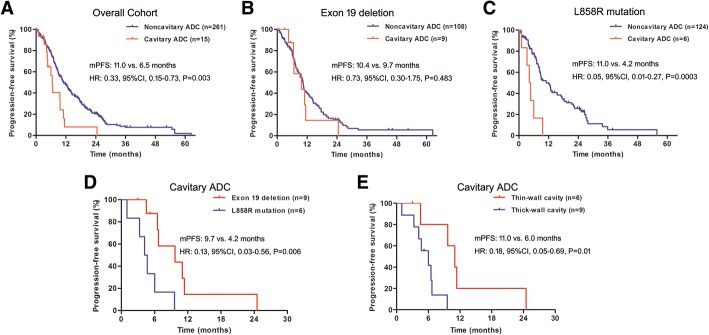
Progression-free survival of first-line EGFR-TKIs in lung adenocarcinoma patients harboring *EGFR* sensitizing mutations. **a** overall cohort; **b** in patients with Exon 19 deletion; **c** in patients with L858R mutation; **d** in patients with cavitary adenocarcinoma according to mutational status; **e** in patients with cavitary adenocarcinoma according to the thickness of cavity wall. Abbreviations: EGFR-TKI, epidermal growth factor receptor- tyrosine kinase inhibitor

The median PFS in patients with noncavitary ADC was significantly better than those with cavitary ADC (11.0 versus 6.5 months, hazard ratio [HR]: 0.33, 95% confidence interval [CI], 0.15–0.73, *P* = 0.003) (Fig. 1a). The ORR for patients who had noncavitary ADC versus those who had cavitary ADC was 59.0% versus 53.3%, respectively (*P* = 0.664), and the disease control rate (DCR) was 93.3% versus 92.0%, respectively (*P* = 1.000).

**Fig. 2:**
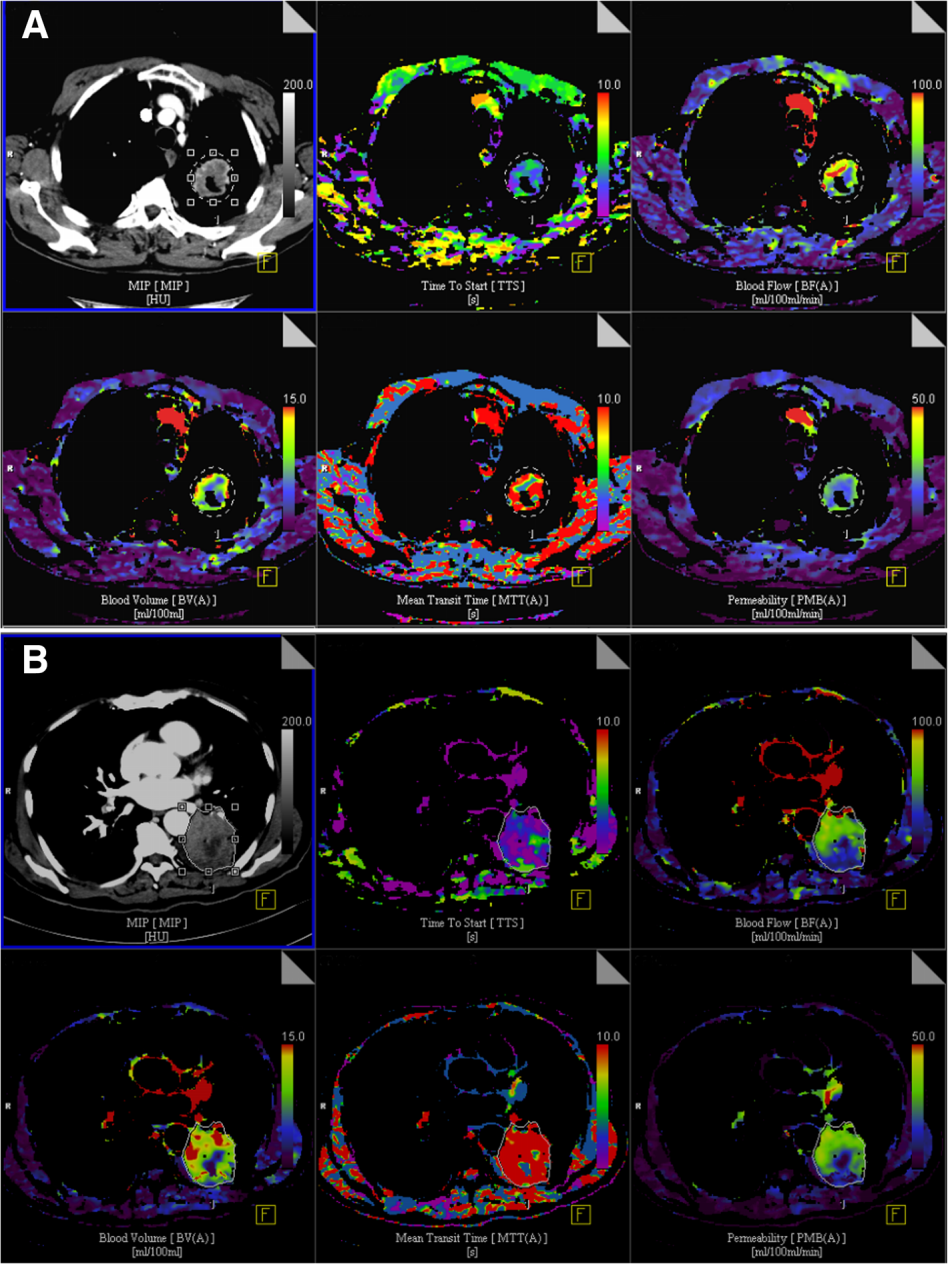
The dVPCT indirectly demonstrated thick-wall cavitary ADC were more hypoxic than non-cavitary ADC. The tumor permeability, blood flow, blood volume and mean transit time was higher in patients with **a** non-cavitary ADC than **b** thick-wall cavitary ADC. Abbreviations: dVPCT, the dynamic volume perfusion CT

We further evaluated the efficacy of EGFR-TKIs in cavitary and noncavitary ADC according to mutational status (19DEL vs. L858R mutation). Interestingly, noncavitary ADC had significantly better PFS than cavitary ADC in patients with L858R mutation (11.0 vs. 4.2 months, HR: 0.05, 95%CI, 0.01–0.27, *P* = 0.0003) while the PFS was not significantly different in patients with 19DEL (10.4 versus 9.7 months, HR: 0.73, 95%CI, 0.30–1.75, *P* = 0.483) (Fig. 1b-c). The ORR (50.0% versus 54.8%, *P* = 1.000) and DCR (83.3% versus 92.7%, *P* = 0.387) were numerically lower in cavitary ADC when compared with noncavitary ADC in patients with L858R mutation. Furthermore, cavitary ADC patient with 19DEL had better PFS than those with L858R mutation (9.7 versus 4.2 months, HR: 0.13, 95%CI, 0.03–0.56, *P* = 0.006) (Fig. 1d). When cavitary ADC was classified according to wall thickness of the cavity, patients with thin-wall cavity had longer PFS (11.0 versus 6.0 months, HR: 0.18, 95%CI, 0.05–0.69, *P* = 0.01) (Fig. 1e), higher ORR (66.7% versus 44.4%, *P* = 0.608) and DCR (100.0% versus 88.9%, *P* = 1.000) than those with thick-wall cavity. The therapeutic responses to EGFR-TKI treatment in cavitary and noncavitary ADC patients with *EGFR* mutations are summarized in Tables 2 and 3. Furthermore, two representative cases showed that noncavitary ADC had higher tumor permeability, BF, BV and MTT than cavitary ADC (Fig. 2a-b).

**Table 3:** A Brief Summary of Responses to EGFR-TKI Treatment in cavitary and noncavitary adenocarcinoma patients with *EGFR* mutations

Characteristic	Cavity *N* = 15	Noncavity *N* = 261	*P*
Response, no. (%)			
CR	0 (0.0)	0 (0.0)	
PR	8 (53.3)	154 (59.0)	
SD	6 (40.0)	86 (33.0)	
PD	1 (6.7)	21 (8.0)	
mPFS (95%CI), mo			
Overall cohort	6.5 (5.3–7.7)	11.0 (9.3–12.7)	0.006
Exon 19 deletion	9.7 (2.1–17.3)	10.4 (9.0–11.8)	0.483
L858R mutation	4.2 (2.5–5.9)	11.0 (7.6–14.4)	< 0.001
ORR, %			
Overall cohort	53.3	59.0	0.664
Exon 19 deletion	55.6	61.1	0.737
L858R mutation	50.0	54.8	1.000
DCR, %			
Overall cohort	93.3	92.0	1.000
Exon 19 deletion	100.0	90.7	1.000
L858R mutation	83.3	92.7	0.387

*Abbreviations: EGFR-TKI* epidermal growth factor receptor- tyrosine kinase inhibitor, *CR* complete response, *PR* partial response, *SD* stable disease, *PD* progressive disease, *mPFS* median progression-free survival, *ORR* objective response rate, *DCR* disease control rate

### Univariate and multivariate analysis on PFS

Univariate analysis identified noncavitary ADC and tumor size ≤5 cm as being significantly associated with better PFS. Multivariate analysis revealed cavity as an independent predictive factor for PFS (HR: 0.49, 95% CI, 0.26–0.90, *P* = 0.022), as well as age (HR: 0.69, 95% CI, 0.49–0.98, *P* = 0.036) and tumor size (HR: 1.74, 95% CI, 1.16–2.62, *P* = 0.008) (Table 4).

**Table 4:** Univariate and Multivariate Cox regression analyses for PFS in Cavitary and Noncavitary Adenocarcinoma patients with *EGFR* mutations

Variables	Univariate Analysis		Multivariate Analysis	
	HR (95% CI)	*P*	HR (95% CI)	*P*
Noncavity vs. Cavity	0.478 (0.271–0.843)	0.011	0.485 (0.261–0.902)	0.022
Female vs. Male	0.846 (0.635–1.127)	0.252	0.793 (0.528–1.190)	0.263
Age > 65 y vs. ≤ 65y	0.790 (0.587–1.062)	0.118	0.691 (0.489–0.976)	0.036
ECOG PS > 1 vs. 0 or 1	0.841 (0.651–1.086)	0.183	0.921 (0.674–1.259)	0.605
Smokers vs. non-smokers	1.124 (0.794–1.591)	0.511	0.919 (0.571–1.480)	0.728
TNM Stage IV vs. stage III + Recurrent	1.022 (0.901–1.160)	0.731	0.972 (0.842–1.122)	0.698
Other EGFR-TKIs^a^ vs. Gefitinib	0.898 (0.666–1.212)	0.483	0.806 (0.574–1.132)	0.214
Tumor size: > 5 cm vs. ≤5 cm	1.886 (1.282–2.775)	0.001	1.741 (1.158–2.619)	0.008
*EGFR* mutation				
Exon 19 deletion	1.000 (reference)		1.000 (reference)	
L858R mutation	1.564 (0.988–2.477)	0.056	1.133 (0.659–1.949)	0.651
Rare mutations^b^	1.339 (0.843–2.127)	0.217	1.140 (0.674–1.928)	0.626

*Abbreviations: EGFR-TKI* epidermal growth factor receptor- tyrosine kinase inhibitor, *PFS* progression-free survival, *HR* hazard ratio, *CI* confidence interval, *ECOG PS* Eastern Corporation Oncology Group performance status

^a^Including erlotinib, icotinib, afatinib (1 patient), AZD9291 (1 patient)

^b^including EGFR mutations in exons 18 and 20, other than 19DEL and L858R mutations

## Discussion

To our knowledge, the current study is the first to investigate the incidence and clinical characteristics of *EGFR*-mutant cavitary ADC and its impact on the therapeutic response to first-line EGFR-TKI therapy. Similar with previous study conducted in overall lung ADC population [14], cavity occurred in 5.4% of patients with *EGFR*-mutant lung ADC and was more prevalent among male patients. Interestingly, our study showed that cavity formation had an adverse impact on the PFS of *EGFR*-mutant ADC treated with first-line EGFR-TKIs. Furthermore, the impaired effect was only observed in patients with L858R mutation but not in those with 19DEL.

Recent evidence suggested patients with 19DEL and L858R mutation may be two distinct subtypes and have different survival outcomes [25]. To date, the possible mechanism remains unclear. In this small fraction of patients, namely *EGFR*-mutant cavitary ADC in our study, we also observed a significantly different PFS among patients with these two subtypes of *EGFR* mutations treated with first-line EGFR-TKIs (19DEL versus L858R: 9.7 and 4.2 months, *P* = 0.006). In the present study, we noticed that all six cavitary ADC patients with L858R mutation had thick-wall cavity (greater than 4 mm) while thick-wall cavity was only identified in one thirds (3/9) of patients with 19DEL. Further analyses showed that patients with thick-wall cavity had worse PFS (6.0 versus 11.0 months, *P* = 0.01) and ORR (44.4% versus 66.7%, *P* = 0.608) than those with thin-wall cavity, suggesting thick-wall cavity may be the main cause that contribute to the worse PFS of first-line EGFR-TKIs in patients with L858R-mutant cavitary ADC. Watanabe et al. previously demonstrated that thick-wall cavitary ADC had poorer survival and a higher frequency of solid predominant tumors, vascular invasion and bronchiolar obstruction than those with thin-wall cavity [18]. Because of the high probability of feeding vessel invasion and bronchiolar obstruction, thick-wall cavity was more likely to result in a hypoxia microenvironment, therefore leading to resistance to EGFR-TKIs. The dVPCT also indirectly demonstrated thick-wall cavitary ADC were more hypoxic, as microvessel density was positively associated with BF and BV and lower BV indicated a more hypoxia status [26, 27]. A previous study also found that patients with solid predominant subtype of ADC harboring *EGFR* mutations had significantly lower ORR (61% versus 88%, *P* = 0.03), shorter PFS (7.7 versus 13.5 months, *P* = 0.002) and OS (21.5 versus 31.0 months, *P* = 0.028) than those with non-solid predominant subtype of ADC [28]. Therefore, the high frequency of solid predominant subtype in thick-wall cavitary ADC may also relate to the poor therapeutic response to EGFR-TKIs in L858R-mutant cavitary ADC. A previous study found that L858R mutation can increase cancer cell invasive ability and malignant pleural effusion formation through activation of the CXCL12-CXCR4 pathway [29]. However, why L858R-mutant ADC tends to promote thick-wall cavity formation needs further investigation.

Another important factor that contribute to the better survival of cavitary ADC patients with 19DEL may be the higher proportion of the T790 M mutation after acquired resistance [30]. In the recent study by Ke et al., they found that patients with 19DEL were more likely to have T790 M mutation than those with L858R mutation when acquired resistance develops (50.4% versus 36.5%, *P* = 0.043) [30]. The median overall survival of patients with T790 M mutation was 36.0 months, which was better than those with other acquired resistance mechanisms, including *MET*-amplification, histology-transformation and *KRAS/PIK3CA/ALK* alterations [30]. In our study, when acquired resistance develops, 25% (1/4) patients with L858R mutation had T790 M mutation while 50% (3/6) in patients with 19DEL. Although the analyzed cases in our study were very small, the incidence of T790 M mutation was in line with previous findings [30–32].

Our study has several strengths including: (1) all patients were newly diagnosed ADC treated with first-line EGFR-TKIs, which ruled out any impact on patients’ outcomes by possible disproportionate pretreatment; (2) all enrolled patients received high-resolution thin-section CT scans (1 mm) in our hospital so that we can accurately evaluate the incidence and characteristics of cavitary ADC. However, our study was also limited in several aspects: (1) it was affected by the limitations inherent to studies with a retrospective design; (2) most of the enrolled patients were diagnosed based on small specimens and ADC subtyping based on small samples is challenging [33–35], so we cannot evaluate whether solid predominant subtype was indeed associated with thick-wall cavity that result in a worse PFS in L858R-mutant cavitary ADC; (3) despite the overall population was large, the number of *EGFR*-mutant cavitary ADC was small, therefore, a large sample study is needed to validate these findings.

## Conclusions

Cavity occurred in 5.4% of patients with *EGFR*-mutant ADC and was associated with a worse PFS of first-line EGFR-TKI therapy, mainly in those with L858R mutation. Thick-wall cavity formation may be the main cause that contribute to the worse PFS of patients with L858R-mutant cavitary ADC.

## Abbreviations

19DEL: Exon 19 deletion
95%CI: 95% confidence interval
ADC: Adenocarcinoma
*ALK*: Anaplastic Lymphoma Kinase.
ARMS: Amplification refractory mutation system
BF: Blood flow
BV: Blood volume
CT: Computed tomography
DCR: Disease control rate
dVPCT: Dynamic volume perfusion CT
EGFR-TKIs: Epidermal growth factor receptor-tyrosine kinase inhibitors
HR: Hazard ratio
*KRAS*: Kirsten rat sarcoma viral oncogene homolog
L858R: Leu858Arg point mutation in exon 21
*MET*: Mesenchymal-epithelial-transformation
MTT: Mean transit time
ORR: Objective response rate
PFS: Progression-free survival
*PIK3CA*: Phosphatidylinositol-4,5-bisphosphate 3-kinase catalytic subunit alpha
QoL: Quality of life
WHO: World Health Organization
